# Single Sequence Whole-Spine Screening Magnetic Resonance Imaging: Diagnostic and Therapeutic Role in Multiple-Level Spinal Tuberculosis

**DOI:** 10.7759/cureus.52757

**Published:** 2024-01-22

**Authors:** Atul Sareen, Mayukh Guha, Kuldeep Bansal, Amit Hegde, Tankeswar Boruah

**Affiliations:** 1 Central Institute of Orthopaedics, Vardhman Mahavir Medical College and Safdarjung Hospital, New Delhi, IND; 2 Spine Surgery, Indian Spinal Injuries Centre, New Delhi, IND; 3 Spine Surgery, Yashoda Super Speciality Hospital, Ghaziabad, IND

**Keywords:** spine biopsy, spinal tuberculosis:, drug resistant spinal tuberculosis, whole spine screening mri, multiple level spinal tuberculosis

## Abstract

Introduction: Spinal tuberculosis (TB) is the most common form of skeletal tuberculosis. Paradiscal continuous vertebral involvement at a single level is the most prevalent pattern among all forms of spinal TB. There is a wide range of reported incidences of multiple-level non-contiguous spinal TB in the literature. We would like to discuss on the utility of single whole spine screening T2-weighted (T2W) mid-sagittal magnetic resonance imaging (MRI) film in diagnosing multiple-level spinal TB and therapeutic benefits it can provide.

Methods: We have done a retrospective review of the collected data of patients in Vardhman Mahavir Medical College and Safdarjung Hospital from August 2017 to October 2021 to find the incidence of multiple-level spinal TB and possible factors attributed to this specific disease pattern. All the patients who had been diagnosed of spinal TB either microbiologically or histopathologically or by a good clinical response to anti-tubercular treatment (ATT) and had a whole spine screening MRI film, were included. Patients of spinal TB who did not have a whole spine screening MRI were excluded from the study. Multiple-level spinal TB was diagnosed when lesions were identified in vertebral levels other than a typical paradiscal lesion, and additional lesions were separated from the primary disease by at least one normal spinal segment.

Results: Among the patients, 242 met the inclusion criteria, and 76 showed multiple-level non-contiguous spinal TB on MRI, incidence being 31.4%. The rest of the 166 patients showed typical single-segment contiguous lesions. By doing multivariate analysis to determine the independent risk factors for multiple-level spinal TB, extremes of age (<20 years and >50 years) have been found to be a significant factor with p value of 0.0001. Though drug resistance was not found to be a significant risk factor (p value 0.051), the proportion of patients having multiple-level TB was far more in the drug-resistant group (13/76).

Conclusions: Single sequence whole spine screening MRI film is an effective, economical, and time-saving tool to detect multiple-level spinal TB. Along with its diagnostic accuracy, it also provides therapeutic benefits like access to a more approachable site for biopsy.

## Introduction

Tuberculosis (TB) has been known to mankind since the days of early civilizations [1]. It is still one of the leading causes of mortality in the world, especially in developing countries [2]. Bone and joint TB contributes to 15-20% of the burden of extrapulmonary TB of which spinal TB is the most common (50%) [3].

Based on the location, spinal TB can be classified as paradiscal, central, anterior, appendiceal, intermediate, and non-osseous types. Paradiscal continuous vertebral pattern is the most common one [4]. One rare form is the multiple-level non-contiguous involvement which is defined as pathological spinal segments (vertebra) separated by at least one normal spinal segment [5]. There is a wide range of reported incidence of multiple-level spinal TB in the literature [6,7]. Also, the incidence is increasing with more detailed evaluation of the patients. Detection of this disease pattern is very important as it has implications for the overall management of the patient.

Radiological investigations play a vital role in the initial detection of spinal TB. Magnetic resonance imaging (MRI) has been able to establish itself as the most powerful imaging modality in identifying spinal TB [8,9,10]. However, a detailed evaluation of all the sequences of the whole spine is time-consuming and very expensive. In our present study, we would like to discuss the utility of single whole spine screening T2-weighted (T2W) mid-sagittal MRI film in diagnosing multiple-level spinal TB and to highlight its advantages in the assessment of patients' treatment.

## Materials and methods

**Figure 1:**
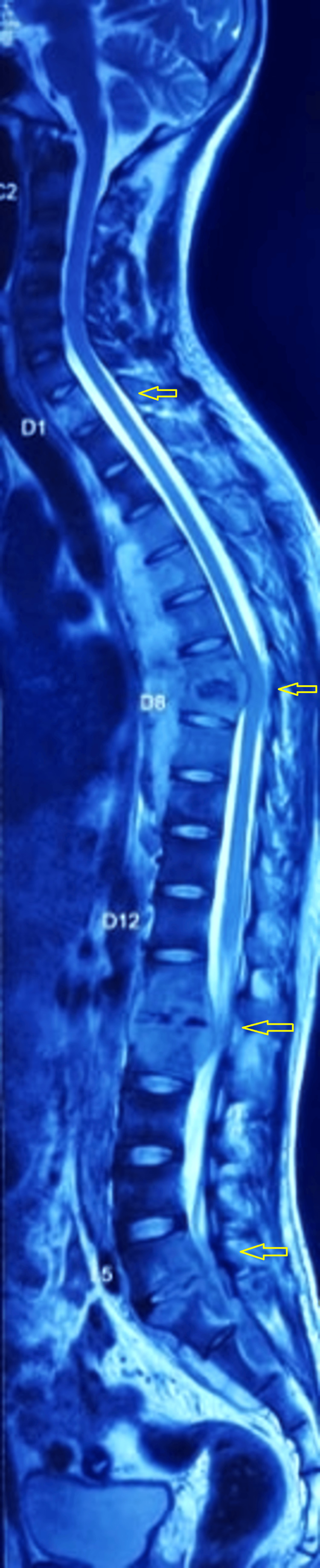
Whole spine T2W screening film showing multiple level TB at D1, D7-8, L1-2, L5-S1 (yellow arrows) TB: tuberculosis; T2W: T2-weighted

From August 2017 to October 2021, we collected clinical, radiological, and other diagnostic data of the patients with spinal TB presented in the outpatient department or emergency of Vardhman Mahavir Medical College and Safdarjung Hospital, New Delhi. We have done a retrospective review of the collected data to find the incidence of multiple-level spinal TB and possible factors attributed to this specific disease pattern. All the patients who had been diagnosed of spinal TB either microbiologically or histopathologically or by a good clinical response to anti-tubercular treatment (ATT) and had a whole spine screening MRI film, were included in the study. The primary site of spinal TB is considered the level for which patients came with complaints and where primary pathology was suspected from clinical examinations. Multiple-level spinal TB was diagnosed when lesions were identified in other vertebral levels, and additional lesions were separated from primary disease by at least one normal spinal segment (Figure 1)

**Figure 2:**
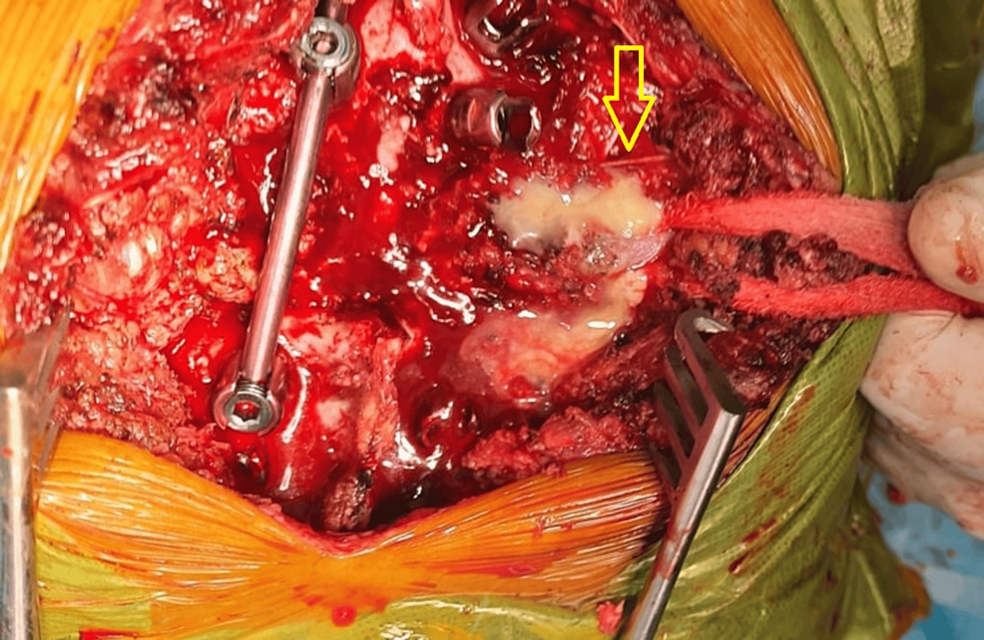
Intraoperative clinical picture showing purulent material coming out from the lesion (yellow arrow)

We have retrieved tissue or pus samples for diagnosis of spinal TB by doing various techniques like computed tomography (CT)-guided biopsy, C-arm image-guided biopsy, ultrasonography (USG) guided aspiration from paravertebral or psoas abscesses, CT-guided fine needle aspiration cytology (FNAC) from paravertebral abscesses according to our convenience and level of the disease. When surgery was indicated, an open surgical biopsy was done (Figure 2).

Tissue samples or aspirated pus were subjected to microbiological investigations like acid-fast bacilli (AFB) stain, AFB culture, aerobic culture, and Gene Xpert. Histopathological analysis of tissue samples was done to look for destruction of osseous trabeculae, epithelioid granulomas with caseous necrosis, and Langhans giant cells. We also sent gram stain, aerobic culture, fungal smear, and fungal culture routinely to exclude other possible causes of spondylodiscitis. For patients in whom tissue diagnosis was not possible, a positive response to ATT was considered diagnostic. For imaging purposes, whenever possible, we ordered a single whole spine screening mid-sagittal T2W MRI film besides the detailed conventional films of the primary suspected lesion.

The statistical analysis was performed using Microsoft Excel (Microsoft Corp., Redmond, WA, USA) and Statistical Package for Social Sciences (SPSS), version 26.0 (IBM Corp., Armonk, NY, USA).

The presentation of the categorical variables was done in the form of numbers and percentages (%). The following statistical tests were applied to the results: (1) The association of the variables which were categorical in nature was analyzed using the chi-square test; if any cell had an expected value of less than 5 then Fisher’s exact test was used; (2) Multivariate logistic regression was used to find out significant factors of multiple-level spinal TB.

For statistical significance, p value of less than 0.05 was considered statistically significant.

## Results

We reviewed a total of 387 patients diagnosed with spinal TB. Out of them, 242 patients met the inclusion criteria of having a whole spine screening MRI. The rest of the patients did not have the whole spine screening film (they either came to us with an already done MRI or didn’t get the whole spine screening MRI done after our advice). Out of the included 242 diagnosed cases, microbiological (AFB culture or Gene Xpert) confirmation was exclusively achieved in 78 cases, 72 cases were exclusively histopathologically confirmed and 45 cases came positive for both. Positive response with ATT was achieved in 47 cases when other diagnostic tests were inconclusive or negative.

Out of 242 cases, 76 patients showed multiple level non-contiguous spinal TB on MRI, incidence being 31.4%. Rest of the 166 patients showed typical single segment contiguous lesions. All the patients showed typical findings of marrow edema, paravertebral collections, subligamentous spread, extradural component, end plate erosion and discitis. Dorsal (66/166) and lumbar (45/166) spine were predominantly involved in single segment disease (Table 1).

**Table 1:** Distribution of spinal involvement in single-level TB (n=166) and multiple-level TB (n=76) patients TB: tuberculosis

Region of spine involved (single-level TB) (n=166)	No of patients (%)
Dorsal	66 (39.76%)
Lumbar	45 (27.10%)
Cervical	29 (17.47%)
Sacral	26 (15.66%)
Region of spine involved (multiple-level TB) (n=76)	
Multiple Dorsal	4 (5.26%)
Multiple Cervical	3 (3.95%)
Multiple Lumbar	3 (3.95%)
Dorsal and Lumbar	28 (36.84%)
Lumbar and Sacral	13 (17.10%)
Cervical and Dorsal	11 (14.47%)
Cervical and Lumbar	7 (9.21%)
Dorsal and Sacral	4 (5.26%)
Dorsal, Lumbar, and Sacral	3 (3.95%)

Among the multiple-level cases, dorsal and lumbar (28/76) involvement was the most common followed by lumbar and sacral (13/76) and cervical and dorsal (11/76). The rest of the cases included cervical and lumbar (7/76), dorsal and sacral (4/76), multiple dorsal (4/76), multiple cervical (3/76), multiple lumbar (3/76) and dorsal, lumbar and sacral (3/76) (Table 1).

The age distribution of patients in both single-level and multiple-level spinal TB is shown in Table 2 and Table 3.

**Table 2:** Age distribution in single-level TB patients (n=166) TB: tuberculosis

Age (years) in single-level TB	No of patients (%)
<=20	28 (16.87%)
21-30	57 (34.34%)
31-40	47 (28.31%)
41-50	15 (9.04%)
51-60	13 (7.83%)
61-70	6 (3.61%)

**Table 3:** Age distribution in multiple-level TB patients (n=76) TB: tuberculosis

Age (years) in multiple-level TB	No of patients
<=20	26 (34.21%)
21-30	15 (19.74%)
31-40	11 (14.47%)
41-50	8 (10.53%)
51-60	11 (14.47%)
61-70	5 (6.58%)

By doing multivariate analysis to determine the independent risk factors for multiple-level spinal TB, extremes of age (<20 years and >50 years) has been found to be a significant factor with p value of 0.0001. Though drug resistance was not found to be a significant risk factor (p value 0.051), the proportion of patients having multilevel TB was far more in the drug-resistant group (13/76) (Table 4).

**Table 4:** Multivariate analysis to determine the independent risk factors for multiple-level spinal TB CI: confidence interval; TB: tuberculosis *p < 0.05

Age (years)	Single-level TB (n=166)	Multiple-level TB (n=76)	Odds ratio (95% CI)	p value
<20 or >50	47/166	42/76	1	0.0001^*^
20-50	119/166	34/76	0.320(0.1818 to 0.5622)	
Drug resistance				
Yes	14/166	13/76	2.240(0.9966 to 5.0363)	0.051
No	152/166	63/76	1	

**Figure 3:**
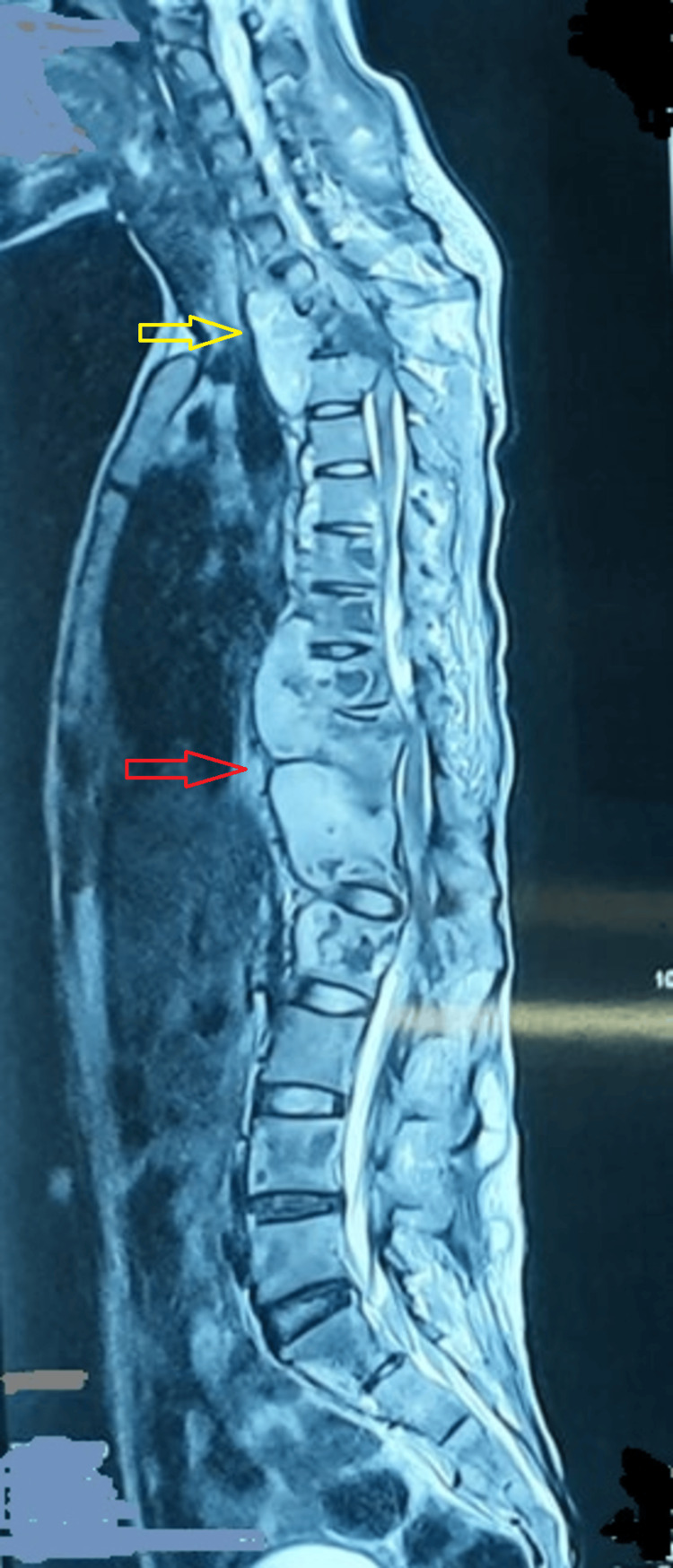
Whole spine screening T2W MRI of a patient with disseminated TB showing extensive involvement of the spine at C7-D3 (yellow arrow) and D7-D12 (red arrow) TB: tuberculosis; T2W: T2-weighted; MRI: magnetic resonance imaging

Furthermore, 2 patients had disseminated tuberculosis on presentation (pulmonary, spinal, abdominal, brain). Both of them had multiple-level skip lesions (Figure 3).

## Discussion

Spinal TB is still a massive burden in our healthcare system in Southeast Asia. It is mainly prevalent in people of lower socio-economic strata in developing countries like India. The numbers are also increasing in developed countries as well due to global migration and increased survivorship of chronic medical diseases and acute immune-deficiency syndrome (AIDS) patients who are immunodeficient [11]. The arterial supply of vertebra is such that the bacteria favor involving either side of the disc and affects subchondral regions of upper and lower endplates [12]. However, besides this typical paradiscal pattern, other patterns of involvement are also seen regularly of which multiple-level non-contiguous pattern seeks special attention. In multiple-level TB, it is possible that the valveless venous plexus is responsible for the disease pattern. This allows the bacilli to reach new vertebrae without being filtered through the lungs or lymphatic system and thus establishing ‘‘skip lesions’’ The other mechanism may be the multiple hematogenous arterial seedings, preferentially targeting different spinal areas, which seems to be less likely [13].

There is paucity in the literature regarding the incidence and management of multilevel spinal TB. Most of them are case reports and reported incidence varied from 1.1% to 71.4% [6,7]. The reason for this may be non-uniform imaging modalities, not doing whole spine screening film routinely, or reporting on the basis of very few cases. Kaila et al. showed the highest incidence (71.4%) but themselves admitted that they have used a very low number of cases and that made the data overestimated [6]. Siddiqui et al. reviewed 187 spinal TB patients and they found the incidence of Non-contiguous multiple level spinal TB to be 25.1% [5]. Polley and Dunn found the incidence of multiple-level spinal TB to be 16.3% in their total set of 98 patients [13]. Batirel et al. reported an 8% incidence of multiple-level non-contiguous lesions in their study [14]. Wang et al. analyzed 597 patients with spinal TB and detected multiple-level skip lesions in 4.19% of cases [15]. We studied 242 patients in a tertiary health care center and found multiple-level TB in 31.4% of cases. So, the actual incidence may very well be much more than already published in literature and we may have to update or modify our treatment protocol in these cases.

There is no doubt about the superiority of MRI in identifying spinal TB. Magnificent contrast resolution helps to identify various patterns of abscesses and multiple skip-level involvement [16,17,18]. Jain AK has stated that MRI has a sensitivity of 100% and specificity of 88% in diagnosing TB spine, well before deformity develops [19]. MRI gives the best idea about the spinal cord status and epidural extension and has the added advantage of perfectly identifying the disease level. Plain X-ray often lags from the actual disease and is only informative once a certain amount of bony damage has been made. CT scans cannot delineate soft tissue and are mainly helpful in assessing the bony status in case of surgical planning. Bone scans and CT myelograms are rarely used nowadays for diagnostic purposes.

In our study, among the single-level TB patients, the maximum was in the age group of 21-40 years (104/166) which is clearly in accordance with the literature we have [4,15,20,21]. We saw that among the multiple-level spinal TB patients, there is a striking trend of affecting the extremes of age; 42/66 patients of multiple-level spinal TB were either below 20 years of age or above 50 years of age (p value 0.0001). This clearly shows that extremes of age have a higher chance of developing multiple-level TB, which may be due to a relatively immunocompromised state. The children, especially the children of lower socio-economic strata of Southeast Asia are often malnourished and immunity deficient, where TB can hit hard and cause multiple-level involvement. Elderly people are prone to diabetes, chronic medical ailments, and sometimes cancer also, making them vulnerable to extensive tubercular infection of the spine.

The incidence of children with spinal multi-drug resistant (MDR) TB is still very much unknown and underestimated in literature [22] Incidence of MDR TB in the geriatric population has also been increased due to possible co-morbidities, presence of underlying chronic ailments, immunosuppression, and malnutrition [23]. In our study, though drug resistance has not been proven to be a significant risk factor (p value 0.051) for developing multilevel spinal TB, the incidence of drug-resistant TB among multiple-level patients (13/76, 17.1%) is almost double that among single-level TB patients (14/166, 8.43%). This clearly shows that drug-resistant TB has a higher propensity to affect multiple levels of the spine and the index of suspicion of drug resistance should be really high among these patients. Also, both of the disseminated TB patients had multiple-level spinal lesions, showing its strong correlation with a severe disease pattern.

Prompt diagnosis of multiple-level spinal TB has big implications in management. It should raise the possibility of an immunocompromised state and necessary investigations should be carried out to rule out any systemic abnormality. It also influences the decision-making about the surgical instrumentation level (whether the skip lesion is to be included within the instrumented level). If not instrumented, then there may be a need for an additional period of bracing to protect the skip lesion segments [5].

Routine use of all sequences of whole spine MRI is not necessary [5]. Only a single mid-sagittal film can delineate skip lesions accurately. This doesn’t put financial stress on the patient and it also saves scanning time. If required later for any reason like surgical planning, we can always go for the other sequences.

Due to the huge patient load, financial constraints, and lack of resources in a developing country like India, obtaining tissue diagnosis is not always possible before starting anti-tubercular treatment in a radiologically suspected case of spinal tuberculosis. Our study clearly shows that the chance of drug-resistant TB is relatively high in multiple-level spinal TB patients. So, a tissue or aspirate diagnosis should be a must to rule out any drug resistance in these multiple-level patients so that a proper anti-tubercular drug regime can be initiated.

**Figure 4:**
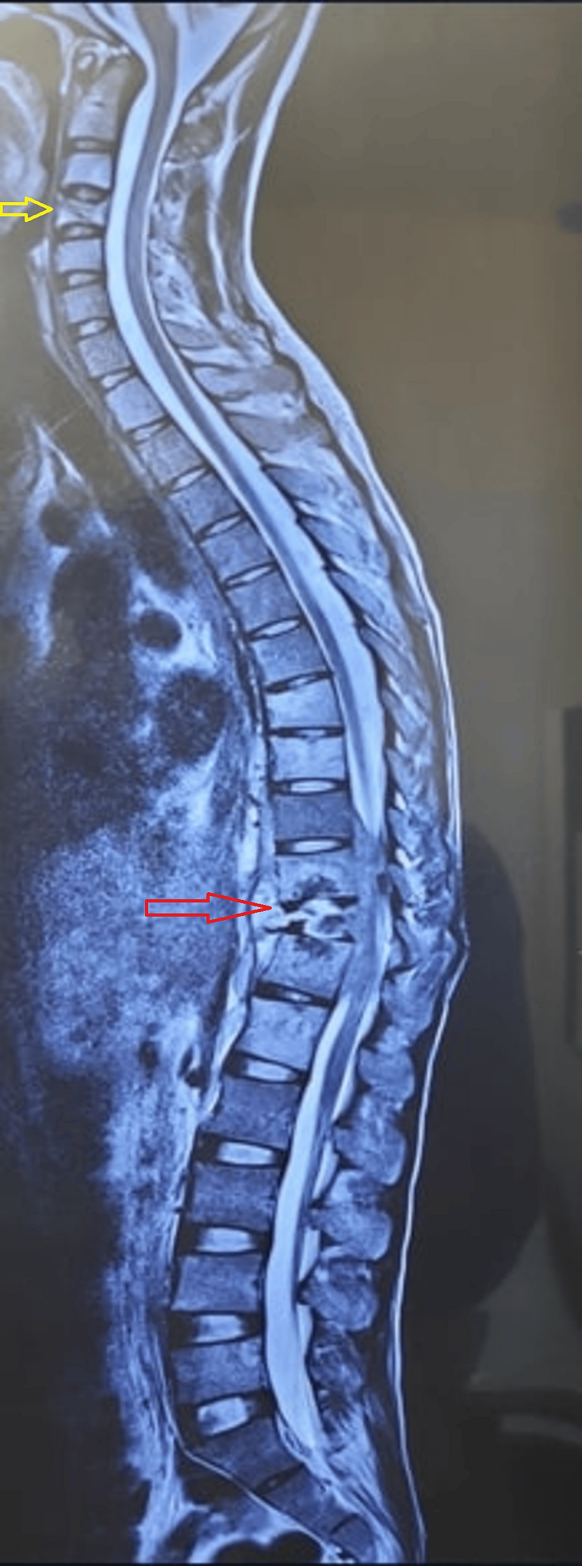
Whole spine screening T2W MRI of a patient who presented with axial neck pain as the chief complaint. MRI revealed multifocal lesions at C4, D7-8, and D10-12. Rather than taking a biopsy sample from C4 (yellow arrow), a transpedicular biopsy was done from the D12 vertebral body and D11-12 disc space (red arrow). This approach was more easy, convenient, and risk-free TB: tuberculosis; MRI: magnetic resonance imaging

Sometimes, in a suspected case of spinal TB, taking a percutaneous biopsy becomes challenging due to location (cervical, cervicodorsal, or high dorsal spine). In whole spine screening film, if skip lesions are found on a more accessible site or segments like mid-dorsal, dorsolumbar, lumbar spine, or sacrum, sample collection becomes easier and biopsy can be planned from that segment before starting any definitive treatment (Figure 4).

The study is not without limitations. Firstly, it’s a retrospective analysis of the previous data. Secondly, we excluded patients who had not undergone a whole spine screening MRI film. So, the actual incidence of multiple-level spinal TB maybe even higher. Thirdly, the incidence rate of multiple-level spinal TB which we found in our study may not clearly define the actual burden of this type of spinal TB in the community. More accurate screening and prospective studies are required to get hold of this.

## Conclusions

The use of single whole spine screening mid-sagittal T2W film can effectively screen multiple level spinal TB patients. The actual burden of these multiple level TB patients is much higher than what is documented in the literature. Single sequence whole spine screening MRI is economical and also less time consuming. It also has therapeutic advantages of having a more accessible biopsy site and giving additional brace protection to a multiple level spinal TB patient whose skip segments are not instrumented in surgery.
